# Discovery of genes and proteins possibly regulating mean wool fibre diameter using cDNA microarray and proteomic approaches

**DOI:** 10.1038/s41598-020-64903-7

**Published:** 2020-05-07

**Authors:** Jinshan Zhao, Huaiyuan Qin, Jingjing Xin, Nan Liu, Rongwei Han, F. M. Perez-Campo, Hegang Li

**Affiliations:** 1 Qingdao Agricultural University, Qingdao, China; 2 Qingdao Scitop Academy of Lactobacillus Co., Ltd, Qingdao, China; 3 Qingdao Institute of Animal Science and Veterinary Medicine, Qingdao, China; 4 China Agricultural University, Beijing, China; 5 University of Cantabria, Cantabria, Spain

**Keywords:** Gene expression, Proteomics, Transcriptomics

## Abstract

Wool fibre diameter (WFD) is one of the wool traits with higher economic impact. However, the main genes specifically regulating WFD remain unidentified. In this current work we have used Agilent Sheep Gene Expression Microarray and proteomic technology to investigate the gene expression patterns of body side skin, bearing more wool, in Aohan fine wool sheep, a Chinese indigenous breed, and compared them with that of small tail Han sheep, a sheep bread with coarse wool. Microarray analyses showed that most of the genes likely determining wool diameter could be classified into a few categories, including immune response, regulation of receptor binding and growth factor activity. Certain gene families might play a role in hair growth regulation. These include growth factors, immune cytokines, solute carrier families, cellular respiration and glucose transport amongst others. Proteomic analyses also identified scores of differentially expressed proteins.

## Introduction

Sheep fleece is a raw material extremely important for the textile industry. Since fibre diameter is one of the most economically significant attributes of sheep wool, the identification of genes regulating this characteristic would offer the opportunity to increase productivity and improve product quality and diversity. This could be achieved by developing breeding programs or through the production of transgenic lines with enhanced characteristics. Besides, drugs can also be develop in order to modify wool fiber properties through gene expression control^1^.

The single nucleotide polymorphisms (SNPs) and quantitative trait loci (QTLs), as well as the molecular mechanisms regulating wool and cashmere growth have been the subject of several studies^1–5^. In mammals, certain gene families, such as those encoding TNFs (tumor necrosis factors), TGFs (transforming growth factors), FGFs (fibroblast growth factors) or proteins involved in WNT signaling, have been previously linked to the development of hair follicle, as well as to hair morphogenesis and cycling^6,7^. Moreover, some molecular aspects of primary wool follicle initiation have been recently reported in Merino sheep^8^.

Microarray and other transcriptome analysis techniques have been successfully employed to analyze the properties of hair follicle stem cells in both mouse^9,10^ and human^11,12^. Different traits, such as resistance to parasites, mammary glands development and milk quality^13–15^, wool follicle development^16^, fleece resistance to rot^17^ or wool and skin pigmentation^18^ have also been studied on sheep and goat using microarrays^19,20^. MicroRNAs putatively involved in goat and sheep hair formation have been identified in the skin of these animals using this approach^21–23^. In addition to microarrays, RNA-sequencing (RNA-seq) has also been used to identify genes differentially expressed in cashmere goat skin during hair follicle initiation and cycling^24^. In fact, the comparative analysis of gene expression in primary and secondary follicles of a cashmere goat, using this technique, identified 51 differentially expressed genes^25^.

RNA-Seq was also recently used to find genes displaying differential expression in diverse sheep tissues^26^, including whole skin, and also between various sheep flocks producing wool with different diameters^27^. Besides, several studies have confirmed the effectiveness of cDNA microarray for establishing the expression profiling of different wool follicle growth stages in whole skin^28–32^.

Mean Wool Fibre diameter (MFD) is one of the main economic traits of wool. To explore the molecular mechanisms regulating MFD, the expression profiles of different stages of embryonic and adult sheep skin have been investigated through scanning of expressed sequence tags (ESTs) and cDNA microarray^16,32,33^. Mutations and epigenetic and post-translational modifications of any ligands or receptors in certain signalling pathways might also influence MFD^1,34^. However, the major pathways regulating MFD still remain unknown.

The Aohan fine wool sheep breed, developed in inner Mongolia, is an excellent sheep breed in terms of meat and wool production. This breed is also highly resistance to disease and to harsh environment and its wool has optimal traits: one animal yields up to 9 kg per year, with a wool length up to 10.5 cm, and a MFD lower than 22μm. Consequently, Aohan fine wool sheep could be regarded as a valuable resource for fine wool production. Only a few works have investigated the genetic characteristics of this particular sheep breed^28,29,32^. The aim of this present work was to explore and compare the gene expression profile of body side skin of Aohan fine wool sheep to its counterpart from small tail Han sheep, a breed producingcoarse wool, during wool follicle anagen phase. We performed both transcriptome and proteome analysis in order to pinpoint the genes and proteins potentially controlling wool diameter in wool sheep.

## Results

### Microarray analysis

All data obtained from the cDNA microarray have been submitted to NCBI’s Gene Expression Omnibus and are available through GEO Series accession number GSE85844 (http://www.ncbi.nlm.nih.gov/geo/query/acc.cgi?acc=GSE85844). 702 probes showed differential expression in the body side skin of the two sheep breeds in the anagen stage (time in August), including 280 probes up-regulated and 422 probes down-regulated in the comparisons of Aohan fine wool sheep versus small tail Han sheep (A/H), as shown in Table S1. Due to a lack of information, the majority of probes (560) were not assigned to specific genes/transcripts. The total number of annotated genes/transcripts was 135, of which 67 were up-regulated and 68 were down-regulated. In A/H, 2 genes, namely LOC443313 and interleukin-8 (IL8) were up-regulated by more than 10-fold, while 5 genes, namely intelectin 2 (ITLN2), 1-acylglycerol-3-phosphate O-acyltransferase 1 (AGPAT1), cytochrome P450 family 1 subfamily A member 1 (CYP1A1), fermitin family member 2 (FERMT2) and LOC101104557, were down-regulated by more than 10-fold.

Several gene families involved in the regulation of different aspects of hair follicle growth displayed differential expression in A/H (see Table S2). These include growth factors, solute carrier families, immune cytokines, cellular respiration and glucose transport among others.

### Validation of microarray results by qPCR

**Figure 1:**
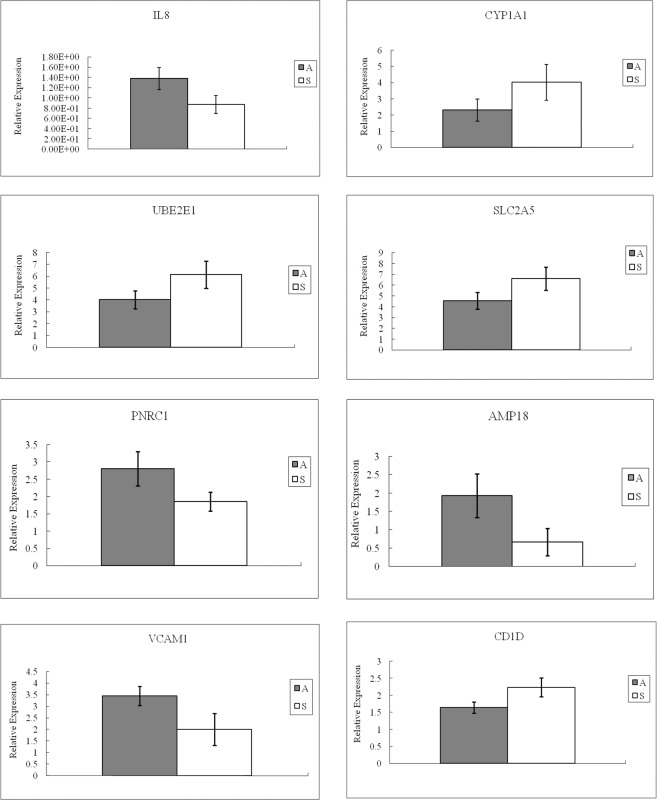
q-PCR validation of the microarray data. *P* values (T-test) of the q-PCR data are 0.005 (IL8), 0.015 (CYP1A1), 0.006 (UBE2E1), 0.006 (SLC2A5), 0.004 (PNRC1), 0.003 (AMP18), 0.003 (VCAM1) and 0.005 (CD1D), respectively. Error bars show the standard errors of the mean estimates.

In order to verify the previous results, we selected 8 differentially expressed (DE) genes, including IL8, cytochrome P450 family 1 subfamily A member 1 (CYP1A1), UBE2E1, SLC2A5, PNRC1, AMP18, VCAM1 and CD1D, to analyse their expression profiles by quantitative PCR (qPCR). The selection criterion for qPCR-validated transcripts is the extent of fold-change of differential expression. IL8 and CYP1A1 were included into the category of high fold-change (>10), while UBE2E1, SLC2A5 and PNRC1 were classified as medium fold-change (3–10), then AMP18, VCAM1 and CD1D were chosen as two representatives of low fold-change category (<3). As illustrated in Fig. 1, the qPCR results for all the genes analysed were in agreement with the microarray results. This clearly highlights the reliability of our microarray data.

### Hierarchical cluster and biological process Gene Ontology (GO) analyses and possible gene networks

**Figure 2:**
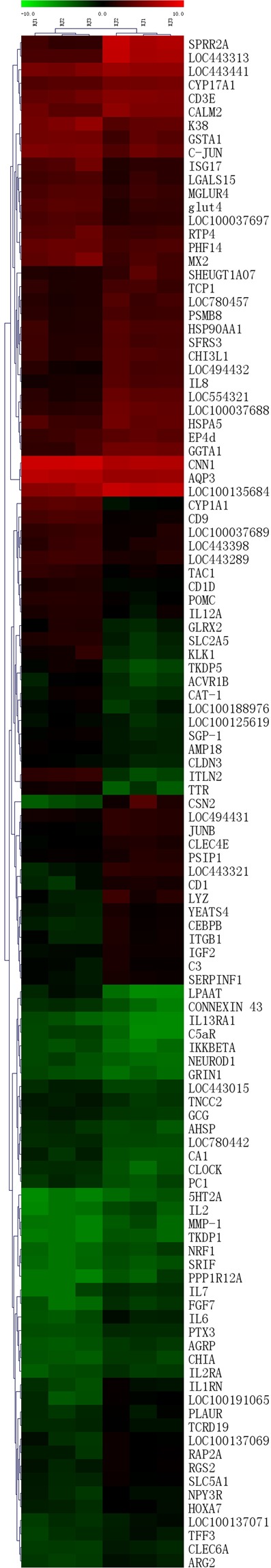
Hierarchical cluster analysis of data between body side skin parts of the Aohan fine wool sheep and small tail Han sheep in anagen phase. Each column represents one sheep, and each horizontal line refers to a gene. Colour legend is at the top of the figure. Red indicates genes with a greater expression relative to the geometrical means, green indicates genes with a lower expression relative to the geometrical means. XJ1, XJ2 and XJ3 represent three repeats of body sideskin of Aohan fine wool sheep; RJ1, RJ2 and RJ3 represent three repeats of body sideskin of small tail Han sheep.Hierarchical cluster analysis of the data indicate that XJ1, XJ2 and XJ3repeats are classified in a tight cluster apparently different from anothercluster containing RJ1, RJ2 and RJ3.

In order to further compare the two sheep breeds in terms of expression patterns of protein-coding genes, we carried out a cluster analysis using the Cluster 3.0 tool. This analysis was able to discriminate the body side skin from Aohan fine wool sheepfrom that of the small tail Han sheep (A/S) (Fig. 2).

**Figure 3:**
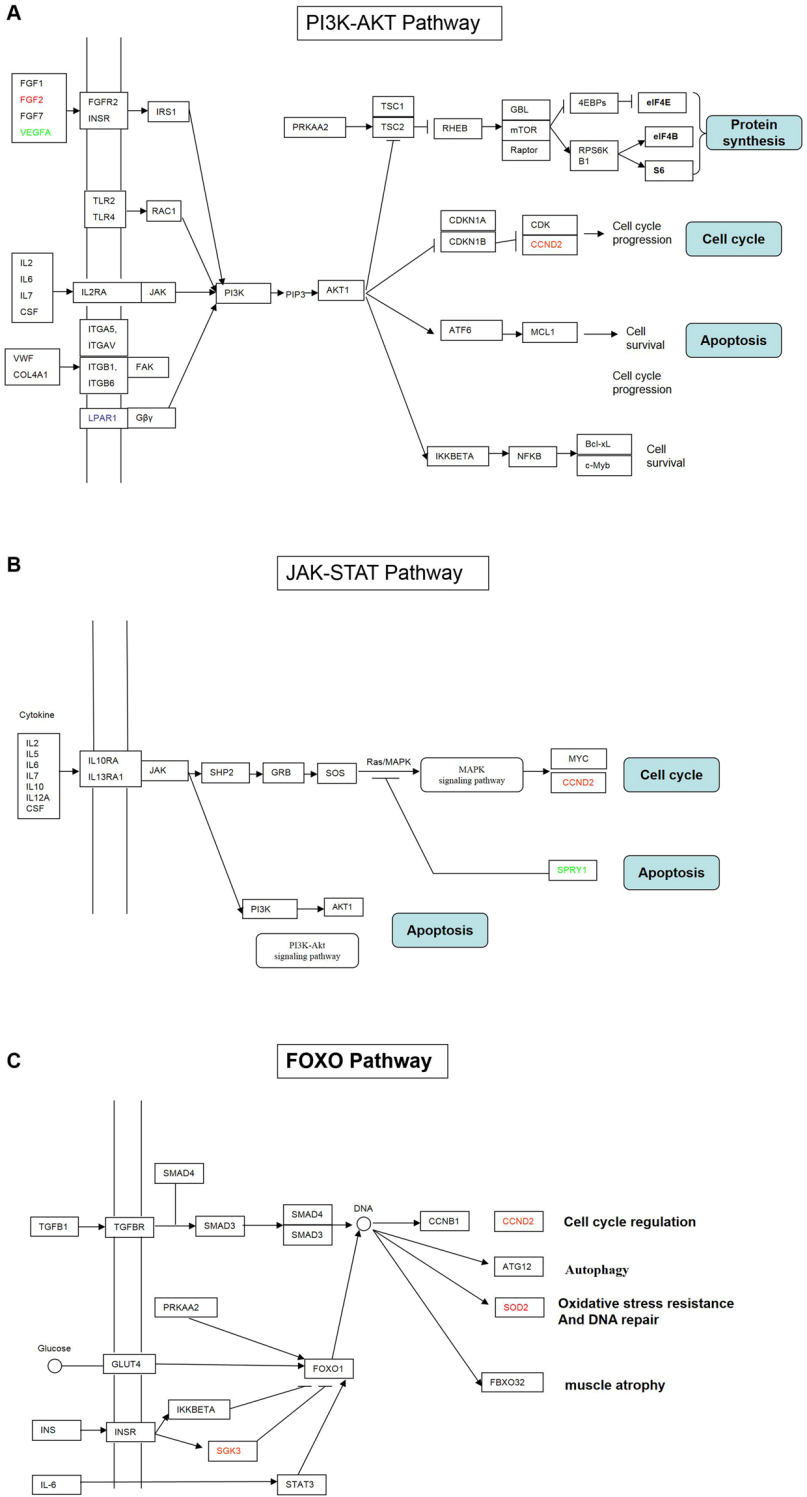
Biological pathways having more differentially expressed genes. (**A**) PI3K-AKT Pathway; (**B**) JAK-STAT Pathway; (**C**) FOXO Pathway. Gene name in red in the gene box indicates higher gene expression in A/S, green indicates lower gene expression, and black indicates no change of the gene expression.

An important number of the differentially expressed genes belonged to three specific signalling pathways: PI3K-AKT pathway, JAK-STAT pathway and FOXO pathway. Figure 3 shows the likely interplays between the differentially expressed genes of these three pathways. These interactions participate in apoptosis and cell cycle.

### Quantitative comparison and protein identification on 2-DE Gels

**Figure 4:**
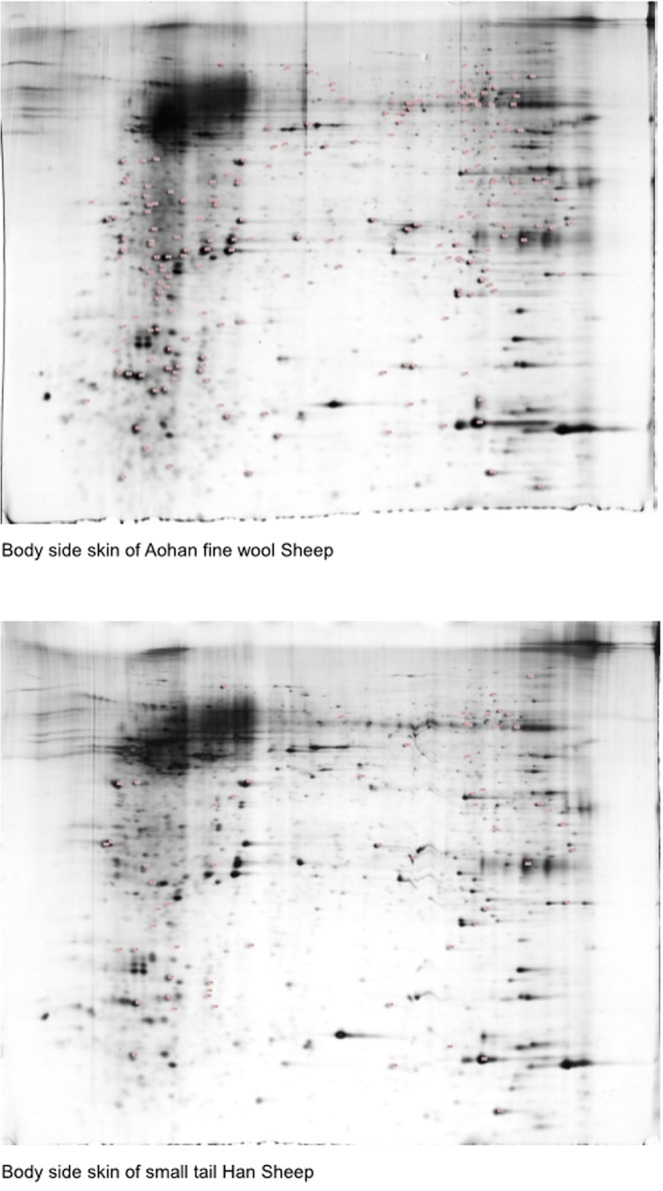
Representative image of 2-DE silver stained polyacrylamide gel.

To pinpoint the differences between body side skins of the two sheep breeds at the protein level, we performed 2D gel electrophoresis with samples for each group in triplicate. Representatives of the results obtained are shown in Fig. 4. Ninety-nine protein spots showed significant differences in terms of expression levels (p < 0.05) between the AS and SS groups. Some of the spots showing significant differences could not be identified by MALDI-TOF/Mass Spectrometry analyses owing to incomplete polypeptide fragments, and some of them were too low in abundance to produce meaningful data. MALDI-TOF/MS analyses allowed the identification of a total of 51 proteins. A list of these proteins including accession numbers and protein/gene names is shown in Table S3. Correlation coefficient between transcriptome and proteome data is 0.1634. Not all the identified DE protein entries were differently expressed at the mRNA level. All MS data have been submitted to Peptide Atlas and are retrievable through Dataset Identifier PASS00797 (http://www.peptideatlas.org/PASS/PASS00797).

## Discussion

### The best time point for identification of major genes determining wool fibre diameter

A reaction-diffusion mechanism controls the distribution, density and size of wool follicles^35–37^ and the size of wool follicle determines, in turn, the wool fibre diameter^35,36^. Wool fibre diameter, as well as follicle density are determinedduringthe initiation of wool follicle^35,36^, therefore primary follicles play more important roles than secondary follicles in determining wool fibre diameter. Since primary and secondary follicle development occur mainly at 50 and 80 days of gestational age, respectively^35,36^, further experiments should include the gene expression profile analysis of foetal sheep skin.

### Analysis of highly differentially expressed genes (>10-fold)

Differential expression analyses performed between the two sheep breed (A/H) showed that 2 genes (LOC443313 and IL8) were up-regulated more than 10 folds in Aohan fine wool sheep.

According to a previous study comparing gene expression in relation to different wool fibre diameters, LOC443313 (type II small proline-rich protein, SPRR) was significantly down-regulated in the super-fine wool group versus the fine wool group^27^. The SPRR proteins constitute a new class of cornified envelope precursors encoded by geneslocated within the epidermal differentiation complex (EDC) region^38^. Two SPRR1, seven SPRR2, one SPRR3 and one SPRR4 genes are found in approximately 300 kb of the EDC^39,40^. These genes are expressed in the epidermis, hair follicles (HFs) and capillaries^41,42^. In our study, LOC443313 was up-regulated more than 10 folds in A/H. This apparent contradiction to previously published data^27^ needs to be further investigated.

IL8 was also significantly up-regulated in the A/D (August vs December)^29^ and S/G (body side skin vs groin skin) during the active growth phase of hair follicles^32^. Human IL8 was also found up-regulated in HF after 4-HC (a chemotherapeutic drug) treatment^43^. The expressionof IL8 gene was induced by the irritants in bulge-derived keratinocytes (BDKs)^44^. IL8 expression was significantly increased in psoriatic hair follicles^45^. Altogether, these results suggest that IL8 might be involved in wool growth regulation. However, whether IL8 plays a role specifically in diameter regulation needs to be determined.

Regarding the genes that were up-regulated, our analyses showed that in A/H, 5 genes were down-regulated by more than 10-fold: ITLN2, AGPAT1, CYP1A1, FERMT2 and LOC101104557.

Expression of sheep abomasal ITLN2 is up-regulated in both mRNA and protein levels after infection with *Teladorsagia circumcincta*^46^.

AGPAT1 promotes the attachment of fatty acyl moieties to the sn-2 position of the glycerol backbone in lysophosphatidic acid during triglyceride biosynthesis^47^. Since fat metabolism and wool synthesis are two processes closely related, down-regulation of AGPAT1 might occur in response to alterations in the barrier lipids of the skin^26^. Interestingly, AGPAT1 was found to be down-regulated in affected tissue of patients suffering from Lichen planopilaris (LPP), a cutaneous disorder that leads to the destruction of hair follicles^48^.

Rowe *et al*.^49^ confirmed that CYP1A1 was predominantly expressed in the sebaceous gland surrounding the hair shaft. CYP1A1 was also down-regulated more than 10 folds in body side skin vs groin skin at both August and December time points^28^ (unpublished data). The expression of CYP1A1 gene was increased scalp biopsies of subjects both unaffected and affected by LPP^48^. Interestingly, CYP1A1 has found to be down-regulated in the body side skin (hair and cashmere rich) of Cashmere goatssubjected to a short photoperiod compared with natural light conditions^50^. Why CYP1A1 expression was also down-regulated over 10 folds in A/H deserves further investigation.

### Analysis of less highly differentially expressed genes (<10-fold)

The gene encoding one cell adhesion molecule, namely vascular cell adhesion molecule 1 (VCAM1) showed differential expression in A/S, suggesting that immune signal transduction had certain universality in the growth processes of wool follicle and wool diameter regulation.

Since different CD antigens are often specifically expressed in different immune cells, so a variety of CD antigens combinations have been used to identify the types of immune cells^12^. Mast cells, macrophages, dendritic cells are involved in immune privilege of hair follicle^51,52^, so the differential expression of CD1D antigen in A/S suggests that it might be associated with immune privilege mechanism.

### Functional implication of differentially expressed proteins

The majority of the differentially expressed proteins found in our study were not mentioned in the previous discussion.

FGF18 protein was down-regulated in A/S. The higher expression of FGF18 in hair follicles is consistent with our studies. Interestingly, this protein is able to induce anagen hair growth when administered subcutaneously to mice in a uniform telogen state^53^.

Keratins are fibrous structural proteins that constitute the structural skeleton of epithelial cells. Keratins are further divided into cytokeratins and hair keratins. KRT1, KRT19 and KRT5 proteins were up-regulated in A/S, while KRT2.13 protein was down-regulated. KRT2.13, as a hair-like gene, was supposed to be inactive in hair follicles^54^. Dowling-Degos disease, characterized by abnormal hair follicles, is a result of at least three different loss-of-function mutations affecting the keratin 5 gene (KRT5)^55^.

Expression of FGFR3 was detected in precuticle cells surrounding the hair bulb^56^. However, in our study, we found that FGFR3 protein was down-regulated in wool bearing skin compared to the control group.

In the skin of foetal Merino sheep, BMP4 mRNA levels quickly raised at the time point of secondary follicle branching^57^. In our study, BMP4 protein was down-regulated in A/S. BMP4 was supposed to play inhibition roles in hair-follicle induction^58^. So BMP4 might play an important role in the process of wool growth.

Stratifin, a protein encoded by the SFN gene thatis required for hair follicle integrity and epidermal homeostasis^59,60^ was up-regulated in A/S. This would further support an essential role of this protein in wool follicle regulation.

### Brief discussion on sampling specificity

In our study, we used two sheep breeds, Aohan fine wool sheep, with fine wool, and small tial Han sheep, with coarse wool, as two models for researching the molecular mechanisms regulating wool fibre diameter. So the main difference between these two sheep breeds is focused on wool fibre diameter. We speculated that the major difference in terms of gene expression profiles between these two breeds was derived from differences at the wool follicles. Certainly, due to the nature of the samples used in this study, we cannot completely exclude that some differences were due to skin tissue outside the wool follicle. But according to the results of a comparative study we conducted at the same time, the differences in gene expression profiles of skin tissues other than hair follicles is relatively small (comparison of gene expression profiles in the hairless region of skin between these two sheep breeds, data not shown). Besides, the fact thatmost of the differentially expressed genes obtained in this study were related to hair follicle development and wool growth regulation underscores our results.

In summary, the data presented here indicate that, at the anagen phase, the wool follicle in the bodyside skin of Aohan fine wool sheep shows a distinct expression pattern when compared to that of small tail Han sheep. Microarray analysis indicated that most of the genes putatively related to wool diameter regulation could be assigned into different categories, including regulation of receptor binding, growth factor activity and immune response. Several gene families might be involved in hair diameter regulation, including growth factors, immune cytokines, solute carrier families, cellular respiration and glucose transport. Proteomic analysis also identified several differentially expressed proteins. This comprehensive study could serve as a starting point of further investigations leading to a better understanding of the molecular mechanisms regulating wool diameter and to the identification of new strategies leading to the production of fine wool.

## Methods

### Animals and sample preparation

Animal handling was performed according to the animal protocols defined by national and local animal welfare bodies. All animal work was approved by the Shandong Province Biological Studies Animal Care and Use Committee^28^.

In August 2010, one male and two female sheep of the Aohan fine wooland small tail Han sheep (one-year-old) breeds respectively, were used in this study. Animals from each breed were half siblingssharing the same male parent. Full-thickness whole skin (including epidermis and dermis) was sampled from the body side skin(more wool growing) of these six animal sunder local anesthesia. These samples were used for both transcriptome and proteome analysis. The skin area sampled was approximately 1 cm^2^. After removal, all samples were immediately stored in liquid nitrogen for RNA and protein extraction^28^. A total of 15, 208 probes encoding proteins were spotted on this Agilent Sheep Gene Expression Microarray (Santa Clara, CA, USA)^28^.

### RNA extraction and microarray hybridization

Total RNA was extracted using TRIzol (Invitrogen). The extraction was performed according to the manufacturer’s protocol^28^. RNA integrity and concentration were evaluated by Nanodrop. Our experiments were accomplished at 2010, when Agilent 2100 Bioanalyzer were not available in China. So we deployed Nanodrop and agarose gel electrophoresis to detect the quality of RNA samples. The hybridization of the RNA samples to the Agilent Sheep Gene expression Microarray (Santa Clara, CA, USA)^28^ was performed by the Kangchen Biotechnology Limited Company (Shanghai, China).

### Microarrays data analysis

After hybridization and washing, microarray slides were scanned with the GenePix 4000B microarray scanner (Molecular Devices, LLC., USA) ^28^. The resulting text files extracted from Agilent Feature Extraction Software (version 9.5.3) were imported into the Agilent Gene Spring GX software (version 7.3) for further analysis^28^. Differentially expressed genes were screened bystudent’s T-test. False discovery rate (FDR) values were generated using permutations of the repeated measurements to estimate the percentage of genes identified by chance^61^.A P valueof0.05 and a FDR value of 0.05 were set as a threshold. Clustering analysis of all differentially expressed genes was performed using Cluster 3.0^62,63^ to analyze the similarity in the expression patterns among different species/breeds^28^. The functional annotation of differentially expressed genes was performed by the DAVID (The Database for Annotation, Visualization and Integrated Discovery) gene annotation tool (http://david.abcc.ncifcrf.gov/)^28,64^.

### qPCR confirmation

Total RNA samples prepared for microarray analysis were also used for qPCR confirmation^28^. Reverse transcription was performed using RevertAid First Strand cDNA Synthesis Kit (MBI Fermentas, Vilnius, Lithuania) according to the manufacturer’s protocols^28^. Primers were designed with the Primer-BLAST program (https://www.ncbi.nlm.nih.gov/tools/primer-blast/)^28^. Primer sequences, melting temperatures and product sizes are described in Table 1.

**Table 1:** Oligonucleotides used for qPCR confirmation.

**Gene**	**Primer sequence (5′-3′)**	**Tm (°C)**	**Target size (bp)**
GAPDH^b^	Forward: GGAGCACGAGAGGAAGAGAGA Reverse: GCCTTGAGGATGGAAATGTATG	60	103
IL8	Forward: GGCCAGGATTCACGAGTTCC Reverse: TCCCGTTTCTCCAAATTCATGC	60	230
CYP1A1	Forward: CAGAGACCACTCTTCCCAGC Reverse: GGGTTCTTCCCCAAGGTCAG	60	259
UBE2E1	Forward: CACCTTCACACCGGAGTACC Reverse: GGTTAGTGCTGGGCTCCAAT	60	133
SLC2A5	Forward: CTACGCAGACCAGATTTACC Reverse: CCATAAGTTCCACCACGA	60	125
PNRC1	Forward: TTTTGGCAGGATTCTGTTTC Reverse: CAGTGACTAGGAGGCTTTGG	60	194
AMP18	Forward: GATAACAACAACAGTGGTGGAA Reverse: TAGATCAGGCTCTTGGGAGG	60	281
VCAM1	Forward: TCAGTTAGAGGATGCGGGAGT Reverse: AGGCGGACGAACAATAGAGC	60	149
CD1D	Forward: GGTATCTGCGAGTAACCCTG Reverse: GACTAAGCCTCCAACAAACAG	60	185

^a^The annealing temperature represents the optimal temperature during quantitative PCR^28^;

^b^RNA levels of GAPDH was assayed for normalization during quantitative PCR^28^.

### Tissue protein extraction

For protein extraction by homogenization, 1% protein inhibitor cocktail and 2% IPG-buffer were added in advance to lysis buffer (42% Urea, 15.2% Thiourea, 4% CHAPS, 1% DTT)^28^. This buffer is then added onto skin tissue previously cut into small pieces by ophthalmic scissors^28^ at the ratio of 1:7 (weight/volume). After tissue homogenization, the tissue slurry is placed at 4 °C for 1 h, vortexing it every 15 min. Following this incubation, samples are centrifuged at 40,000 g for 30 min. Supernatant is separated and store it at −80 °C. Protein concentration was determined by the Bradford method.

### One-dimensional electrophoresis

Add 0.5% IPG-buffer into 150 μg protein sample (400–600 μL), and place it into One-dimensional electrophoresis instrument^28^. The programme is as below:

Step-n-hold

S130 V 6 h

S2 60 V 6 h

Gradient

S3 500 V 1 h

S4 1000 V 1 h

S5 3000 V 3 h

S6 8000 V 3 h

Step-n-hold

S7 8000 V 20 h

### 2-dimensional (2-D) SDS-PAGE preparation

Tris-HCl (PH = 8.8), Monomer storage (30% Acrylamid and 0.8% NN′-methylenebisacrylamid), 10×electrophoresis buffer (3.03% Tris-Base, 14.4% Glycine, 1% SDS), balanced solution (36.05% Urea, 5% Tris-HCl, 2% SDS, 34.5% Glycerine)^28^.

The electrophoresis programme used for the second dimension SDS-PAGE is as follows^28^:

Transfer: Voltage 300 v, Current 50 mA, Time 1 h.

Separation: Voltage 300 v, Current 200~250 mA, Time 4~5 h.

Fixative preparation: 40% Ethanol and 10% Acetic acid.

Fixation: take out the rubber strip and put it into Fixative for 1 h.

### Staining and comparison of expression levels

Gels were silver stained, scanned and analysed using Image Master TM 2D platinum software (Version 5.0, GE Healthcare, San Francisco, CA, USA)^28^. The expression level was determined by the relative volume of each spot in the gel and expressed as %Vol (%Vol = [spot volume/Σvolumes of all spots resolved in the gel])^28^. We calculated the means and standard deviations of both sample groups and assessed statistical significance with Student’s t-tests using Image Master TM 2D platinum software^28^. P values < 0.05 were considered statistically significant^28^.

### Identification of altered proteins by mass spectrometry (MS)

Protein spots with significant differences between the two groups were excised, dehydrated in acetonitrile, and dried at room temperature^28^. Gel pieces were denatured, alkylated, trypsin digested and analysed by an Ultraflex II MALDI-TOF-TOF mass spectrometer (Bruker Daltonics GmbH, Bremen, Germany) under the control of FlexControl TM 2.4 software (Bruker Daltonics GmbH)^28^. Acquired peptide mass fingerprint (PMF) were processed using the software Flex Analysis^TM^ 3.0 (Bruker Daltonics, Bremen, Germany)^28^. The peak detection algorithm was: SNAP (Sort Neaten Assign and Place); S/N threshold: 1.5; Quality Factor Threshold: 50. The tryptic auto-digestion ion picks (trypsin [108–115] 842.5094 Da, trypsin [58–77] 2211.104 Da) were used as internal standards^28^. The resulting peptide mass lists were used to search the Matrix science database (http://www.matrixscience.com)^28^. The following search parameter criteria were used^65^: mass tolerance 100 ppm, miss cleavage ≤ 1, modification comprises Carbamidomethyl and methionine oxidation^28^. Matched peptides number between experimental PMF and theoretical PMF ≥ 5^28^.

## Supplementary information

Supplementary information

Supplementary information2

Supplementary information3
