# Supplementary material for: Discovery of genes and proteins possibly regulating mean wool fibre diameter using cDNA microarray and proteomic approaches
Source: Sci Rep. 2020 May 7;10:7726. doi: 10.1038/s41598-020-64903-7 (PMC7206055; doi:10.1038/s41598-020-64903-7)
Supplement: Supplementary file 2 — Supplementary information2 [file 41598_2020_64903_MOESM2_ESM.pdf]

Exploring differentially expressed genes and proteins in the skin of Chinese fine and coarse wool sheep breeds using cDNA microarray and proteomic approaches

Jinshan Zhao[1,2,3,4,*] , Huaiyuan Qin[1,*], Jingjing Xin[1,*], Nan Liu[1], Rongwei Han[1], FM Perez Campo[5], Hegang Li[1,2,3]

[1] Qingdao Agricultural University
[2] Qingdao Scitop Academy of Lactobacillus Co., Ltd
[3] Qingdao Institute of Animal Science and Veterinary Medicine
[4] China Agricultural University
[5] University of Cantabria

***These authors contributed equally to this work.**
Correspondence and requests for materials should be addressed to H.L.(email: lihegang2000@aliyun.com)

Table S2 DE genes and their relation to different aspects of hair follicle growth [1]

Factor Family	Gene description	probe set ID	Fold Change
	Growth and transcription factors		
Fibroblast growth factor (FGF)			
FGF2	Ovis aries fibroblast growth factor 2 (basic) (FGF2)	A_70_P039492	1.93
Vascular endothelial growth factor (VEGF)			
VEGFA	vascular endothelial growth factor A	A_70_P061521	-1.88
Other transcription factors			
LOC494431	Ovis aries transcription factor YY1 mRNA, partial cds. [AY656760]	A_70_P003261	1.92
TFAP2A	Ovis aries transcription factor AP-2 alpha (activating enhancer binding protein 2 alpha) (TFAP2A), mRNA [NM_001009745]	A_70_P039521	1.64
ETS2	Ovis aries transcription factor Ets-2 mRNA, partial cds. [AF057716]	A_70_P049396	1.37
CREBZF	PREDICTED: Ovis aries CREB/ATF bZIP transcription factor (CREBZF), mRNA [XM_004019767]	A_70_P002046	-2.37
	Cytokines		
VCAM1	ovine_partialCDS_VCAM1 texel sheep partial heterologous PCR in lymph node Ovis aries cDNA similar to ovis aries Vascular cell adhesion molecule 1, VCAM1, mRNA sequence [ES414810]	A_70_P059931	1.98
	Ubiquitination		
UBE2E1	PREDICTED: Ovis aries ubiquitin-conjugating enzyme E2E 1, transcript variant 1 (UBE2E1), mRNA [XM_004021822]	A_70_P059781	-3.83
LOC101122127	PREDICTED: Ovis aries ubiquitin-40S ribosomal protein S27a-like (LOC101122127), mRNA [XM_004015103]	A_70_P014911	1.50
	Enzymes		
Cytochrome P450			
CYP17A1	Ovis aries cytochrome P450, family 17, subfamily A, polypeptide 1 (CYP17A1), mRNA [NM_001009483]	A_70_P012531	2.27
CYP1A1	Ovis aries cytochrome P4501A1 (CYP1A1), mRNA [NM_001129905]	A_70_P041841	-14.77

	CD antigens		
CD1D	Ovis aries CD1d molecule (CD1D), mRNA [NM_001123001]	A_70_P002727	-2.77
	Cyclin dependent		
CDK1	Ovis aries cyclin-dependent kinase 1 (CDK1), mRNA [NM_001142508]	A_70_P047446	1.55
CDKN1C	Ovis aries cyclin-dependent kinase inhibitor 1C (p57, Kip2) (CDKN1C), mRNA [NM_001142510]	A_70_P004976	-1.56
CCND2	020605ONLN034043HT ONLN Ovis aries cDNA, mRNA sequence [EE828214]	A_70_P005691	1.25
	Solute carrier families（SLCs）		
SLC2A5	Ovis aries solute carrier family 2 (facilitated glucose/fructose transporter), member 5 (SLC2A5), mRNA [NM_001009451]	A_70_P051037	-7.17
SLC35F2	PREDICTED: Ovis aries solute carrier family 35, member F2 (SLC35F2), mRNA [XM_004015996]	A_70_P032446	-5.64
SLC25A4	Ovis aries solute carrier family 25 (mitochondrial carrier; adenine nucleotide translocator), member 4 (SLC25A4), mRNA [NM_001127278]	A_70_P011181	-1.44
SLC38A2	PREDICTED: Ovis aries solute carrier family 38, member 2, transcript variant 2 (SLC38A2), mRNA [XM_004006422]	A_70_P043642	6.00
	Cellular respiration		
SLC25A4	Ovis aries solute carrier family 25 (mitochondrial carrier; adenine nucleotide translocator), member 4 (SLC25A4), mRNA [NM_001127278]	A_70_P011181	-1.44
ATP6V1B2	PREDICTED: Ovis aries ATPase, H+ transporting, lysosomal 56/58kDa, V1 subunit B2 (ATP6V1B2), mRNA [XM_004004217]	A_70_P031221	-1.68
	Glucose transport		
SLC2A5	Ovis aries solute carrier family 2 (facilitated glucose/fructose transporter), member 5 (SLC2A5), mRNA [NM_001009451]	A_70_P051037	-7.17
GLUT-1	Ovis aries glucose transporter type 1 (GLUT-1) mRNA, partial cds. [U89029]	A_70_P026062	-1.97

References

1 Stenn, K. S. & Paus, R. Controls of hair follicle cycling. *Physiol Rev* **81**, 449-494 (2001).
